# Unchallenged good intentions: a qualitative study of the experiences of medical students on international health electives to developing countries

**DOI:** 10.1186/1478-4491-12-49

**Published:** 2014-09-04

**Authors:** Patrick O’Donnell, Eilish McAuliffe, Diarmuid O‘Donovan

**Affiliations:** Graduate Entry Medical School, University of Limerick, Limerick, Ireland; Centre for Global Health, Trinity College, University of Dublin, 7-9 Leinster Street South, Dublin 2, Ireland; Clinical Science Institute, National University of Ireland Galway, Room 342, Galway, Ireland

**Keywords:** Medical student, International health elective, Developing country

## Abstract

**Background:**

Irish medical students have a long and proud history of embarking on international health electives (IHEs) to broaden their experience in the developing world. Although there are many opinions in the literature about IHEs, there is a dearth of empirical research that explores the experience and the value of these experiences to medical students. Most students who participate in these IHEs from Irish medical schools are members of student IHE societies, which are entirely run by students themselves. There are varying levels of preparation and interaction with the medical schools in planning these experiences. This study explores the experiences of a sample of students who completed IHEs in 2012.

**Methods:**

This qualitative study used anonymized one-on-one interviews with medical students in Irish medical schools who completed IHEs to developing countries in 2012. Students were recruited using online noticeboards of student societies and e-mail. Purposive sampling was used to find students from different medical schools, some who had travelled with medical student IHE societies and others who had travelled independently. Seven male and seven female students participated. Interviews were conducted until saturation was reached. Data were then analyzed thematically.

**Results:**

The main themes identified were the perceived benefits of IHEs, the difficulties experienced with the distribution of charitable donations, the emotional impact on the students of participating in the IHEs, awareness of scope of practice by students, and issues with the current structure of IHEs.

**Conclusions:**

The informal relationship that currently exists between student societies and the medical schools results in poor accountability and reporting requirements on IHEs. Clearer guidelines and identification of learning outcomes for students would be helpful. The findings are relevant to medical students internationally.

## Background

There is a long history of medical students travelling to developing countries to further their understanding and experience of health care. In Ireland, where we have a long and proud history of medical missionaries going to low resource settings, medical students have been taking part in international health electives (IHEs) for over forty years. Five of the six medical schools in Ireland have medical student IHE groups or societies which organize students for the experience. These societies are run entirely by students themselves and membership of them is typically open to students in the third year of studies. Their main role is to help fundraise for donations to the IHE host institutions in developing countries. Most students who travel to low resource settings on IHEs are members of these societies, and a small number of others travel independently.

In Ireland, there is a certain level of recognition of IHEs by medical schools, but there is a lack of consistency in the approach to them. Some medical schools offer elements of pre-departure training, while others formally recognize time spent away by awarding academic credit. IHEs tend to last three to four weeks. Students generally travel after completing third or fourth year of medical school. For some, they will not yet have entered the clinical years of medical training. Many visit hospitals or clinics that have previously hosted Irish IHE students. Preparations prior to embarking on IHE tend to be limited to fundraising and obtaining travel vaccinations. These are mainly arranged by the students themselves or their IHE groups. There is no formal register of students who go on IHE annually, so figures for the number of medical students who participate are estimates gathered from student IHE groups. Here in Ireland there are no specific guidelines on student conduct while on IHE; this compares with the UK where specific standards are set out by the General Medical Council (GMC) to advise students of appropriate behaviour while on elective [1]. The employment of private companies who specialize in arranging IHEs for students is not common in Ireland.

Following a review of the relevant literature it is understood IHEs are considered a very important part of undergraduate medical education and as an editorial in *The Lancet* explained: ‘Nothing else in the undergraduate curriculum is quite like it or could replace it. No other part of the course transforms students so rapidly and profoundly’ [2]. Authors such as Ackerman and O’Neill have extolled the virtues of using well-planned IHEs as a tool for teaching about caring for disadvantaged groups [3, 4]. Others have commented that while medical curricula and evaluation of these have been enhanced and developed over the years, IHEs have not been similarly placed under the microscope [5, 6]. A positive development in this field has been the publication of a report by the Working Group on Ethics Guidelines for Global Health Training (WEIGHT) [7]. These recommendations acknowledge the huge increase in interest in global health and IHEs in medical schools. The authors discuss the benefits of IHEs, but also note the possible difficulties that can arise due to these electives.

Elit *et al.* conducted a qualitative study that highlighted some of the problems that can arise on IHEs [8]. Students described having great difficulty in knowing who to try to help in the face of so much need and deprivation in developing countries. This left some of them feeling upset and deflated after their elective.

Most other studies focus primarily on self-reported outcomes, such as improvements in clinical skills and cultural awareness [9, 10]. A lack of standardization in all aspects of the running of IHEs hampers comparisons between medical schools. What these types of evaluations do not consider are the wider issues for participating students. They do not capture the essence of what it means to be part of these often life-changing experiences. Factors such as how their experiences affect them while away and on their return, are often ignored. This study attempts to address this gap by undertaking a qualitative study with recent participants of IHEs and discusses their experiences in depth. By engaging in interviews with students and recording their experiences and insights, this study raises some issues for medical schools and establishes a platform for further research.

## Methods

### Approach and research design

One-on-one, semi-structured interviews were conducted in person to record the experiences of the participating students. A phenomenological approach was adopted. Approval was granted by the Research Ethics Committee of the Centre for Health Policy and Management and the Centre for Global Health, Trinity College, University of Dublin. The principal investigator (POD) contacted student groups in each of the medical schools to advertise for the research study on online noticeboards and on their student e-mail contact lists in order to recruit potential participants. Some of these groups were general medical student societies, and others were specific medical student IHE societies. The inclusion criteria for the research study were that each participant had to be a medical student attending a medical school in the Republic of Ireland who had undertaken an IHE in a hepatitis A endemic country in 2012 [11]. Endemic levels of hepatitis A infection have been used in the past as a marker for developing country status [12].

The responses to the advertisement were screened and participants purposively selected and invited to participate in a qualitative interview. The screening was to ensure the inclusion criteria were met (that is they had travelled in 2012 and they had been in a suitable country), and also to discover what medical school the student was attending and whether they were part of a student group or travelled independently. The different medical schools in Ireland have different approaches to IHEs, with some offering basic preparation and others none. Medical student IHE groups do make some pre-departure arrangements, so it was felt that students inside and outside these groups should be included.

Informed consent was gained from each participant. In the latter stages of the research project, snowball sampling was used to reach students from one medical school that had not yet been represented in the study. In the final study population there were seven male and seven female participants, with three who had entered medical school as postgraduates. Five of the six medical schools were represented. Two students travelled independently of student IHE groups. All of the participants had been on IHE in the summer of 2012. The research interviews took place in the summer of 2013, a year later. It was hoped this time lag would allow a period of reflection by the students who had been on IHE.

The researcher himself had personal experience of undertaking three IHEs as a medical student. One of these trips was as part of a student IHE group, one was independently and the last was with a university charity with a focus other than health care. The status of the researcher as a qualified doctor and a postgraduate student of global health was made known to the participants prior to each interview, but it was made clear that he was conducting this study as an impartial researcher.

### Data collection and analysis

An interview topic guide was utilized by the researcher. This covered the reasons for going on IHE, the expectations of the student, plans for before during and after the IHE, feelings on the IHE in hindsight and advice for future IHE participants. This was derived from the researcher’s own experiences of IHEs and a review of the relevant literature. Each interview was voice recorded. All information was anonymized during the transcription process. Thematic analysis was carried out as described by Braun and Clarke [13]. NVivo (Version 10; QSR International Pty Ltd, Doncaster, Victoria, Australia) computer software was used to make the coding process more efficient. An audit trail of all coding, revisions and models of themes and subtheme arrangements was maintained so the research process was reliable and traceable [14, 15]. Interviews were conducted and analyzed sequentially until saturation was reached. Fourteen interviews were conducted from April to June 2013. Interviews lasted on average 55 minutes. Triangulation of data by having different researchers code the data was not possible due to time and resource constraints. Lincoln and Guba urged readers to consider the ‘transferability’ of qualitative research findings, and to be able to make that assessment in this case clear information on the study participants and setting has been provided [16]. They also explained the concept of confirmability which is shown when the researcher involved acknowledges and ‘brackets’ their prior experience and knowledge of the topic under investigation. The researcher in this case was acutely aware of his experiences on IHEs from the outset. During analysis, equal attention was given to dissenting or differing viewpoints of participants in the data. The ‘COnsolidated criteria for REporting Qualitative studies’ (COREQ) was consulted to help formulate a concise report of this qualitative study [17].

## Results

Five main themes emerged and these are presented in turn below.

### Perceived benefits of IHEs

Students reported many benefits from taking part in IHEs. Some of these included learning about new cultures and health systems functioning in low-income countries:

‘Culturally, it was fascinating and I feel like I learned a lot about their health systems.’ P11

Others mentioned they had improved their communication skills, their clinical skills and that they now had experience with diseases that were previously unfamiliar to them. Some also complimented the standard of teaching received from medical staff in the host setting.

### Difficulties with the distribution of charitable donations

As explained above, the majority of students taking part join IHE student societies. While these organizations have many roles in the running of IHEs, the major activity is fundraising. Much planning and organization goes in to raising the money for donation to the host clinics, but there is little direction on how to administer these funds once the students reach the developing country. The total amount raised by one student IHE society in the year being researched was €125,000, and this was then divided between the participants before departure.

Some of the student IHE societies advised travelling students not to reveal to local medical staff that they had brought money to donate, for fear it would be misappropriated:

‘We tried to kind of bypass the kind of head of the hospital because he’s meant to be a little corrupt, so when we got there we didn’t actually mention anything about money to him. I think he expected us to have money but we tried to, because the students before us would have, we tried to bypass him and kind of just donate it straight into the funds as oppose to letting him get hands on it.’ P8

Having funds to donate placed the students in a position of relative power, and how they dealt with this varied from group to group. For one group, they were able to provide the entire medicines budget for the hospital for a year by paying €18,000 directly to pharmaceutical companies, rather than giving to their hosts and risk it being incorporated into the hospital budget and diverted elsewhere. In another case, the group developed a monitoring scheme for the construction of a hospital ablution block, whereby they would donate for the construction in instalments long after their return to Ireland:

‘That’s still being built and they are still e-mailing us pictures because we have to see it going up … We only put in 10% of the money each time. The money goes in as they do it; it’s just being finished now.’ P9

Behind all of these precautions was the issue of accountability; accountability to the student elective group at home, but also to the public who had so generously donated funds.

Some students took the view that donating money was a type of transaction:

‘I think that it’s a symbiotic relationship between … ourselves and the hospitals because we bring over funds, and we hope that that’s helping them. They, in return, give us an educational experience … There were students who went over there, and didn’t bring anything, any money or anything. I thought that was sad, I think it was an attitude to take, take, take and I liked that we we’re giving something back.’ P7

The issue of the host facilities and hospitals becoming dependent on these student groups was raised in a number of interviews. Some felt that the hospitals were desperate for the donations they received annually and this was seen in pleading communications to the students in the months before they embarked on IHE:

‘They were getting e-mails saying: “when are you coming out? We’re very low on funds” … So they were almost being pressurized for the money before they were even there … They’re becoming dependent on the money coming in every year.’ P8

### Emotional impact on students

Much of the existing literature extols the many benefits of IHEs for students. The personal struggles some students face while away and the doubts they can have after returning home are rarely mentioned in the literature. One student described the difficulties he faced when seeing a child he suspected of having pulmonary TB at a rural medical clinic. He was advised by the clinical officer to prescribe a short course of antibiotics as there were no investigations available for TB:

‘I found that hard to deal with … I knew I was doing something incorrect, but I was told to so, that was hard to deal with.’

Researcher: ‘Have you thought about some of those things since?’

‘Yeah a lot and I go, “uh oh”.’

Researcher: ‘What do you think about them now when you reflect?’

‘I hope the kid is still alive, that I didn’t do harm, even though I wasn’t able to help them.’ P1

He expressed his misgivings further by saying:

‘I felt sometimes that we were practicing very bad medicine.’ P1

The use of insufficient amounts of anaesthetic and analgesia for surgical procedures was a frequent topic of concern raised by the students. One participant described witnessing a major surgical procedure:

‘99.9% of the people here would call it barbaric, but I think over there, because the surgeon and the two medical officers were accepting it, then I didn’t really know what to say.’ P7

A number of participants had broached the subject of perceived inadequacies in the medical care with their local supervisors while they were on the IHE. In many developing countries clinical officers are employed to provide care when doctors are unavailable. This student had complained about one of these clinical officers:

‘She said, “well you know, he’s not a doctor, and you have to make allowances for the lower level of training here and everything”. Just kind of I was encouraged that it wasn’t worth it to get into it with him, because he was really the only one there who had some degree of experience in that area, and they needed him.’ P10

A number of participants commented that the research interview for this study was the first opportunity they had had to discuss these troubling episodes.

### Awareness of scope of practice and boundaries

Some students had very firm boundaries with regards to carrying out activities beyond the scope of prior training. Others did carry out new activities and procedures with encouragement and supervision of host medical staff.

One student articulated his feelings on the differences between volunteering and going on IHE as follows:

‘I think it’s quite misguided to call yourself a volunteer if you don’t have the required skills … after third med I did have some skills gained in hospitals here but, obviously I am nowhere close to a doctor … I wanted to be sure that I wasn’t going over there on my high horse, saying, “oh look at me, I’m coming from Ireland, I have a university education”’ P10

Another student who was involved in the student IHE group planning and describes the advice he gave others before leaving home:

‘If you are uncomfortable about doing something or you don’t know how to do something, don’t do it just because someone’s asked you to do it. Don’t do it because you think it’d be a cool learning experience if you actually don’t know how to do it.’ P12

There were some cases where students acknowledged they had acted beyond their level of prior training, and they went on to justify these situations. The first explained that there had been an ‘all-out’ strike by doctors and the IHE students were the only ones left supporting a consultant at a busy hospital:

‘I suppose there’s an ethical issue of us actually taking the role of almost a doctor, which in a way we did … That was definitely wrong, we didn’t know enough to be doing it, but at the same time I suppose, we were being supervised and there wasn’t really another choice.’ P4

Another student who felt he had been practicing above his level of training explained:

‘The clinical officer was happy to have us do it … It’s a setting of such limited resources I guess, it changes how much a medical student should do and it’s either that or absolutely nothing at all. But, yeah I wouldn’t be allowed to do that in Ireland.’ P1

This is the crux of the matter - does the developing world setting mean a medical student should take on more responsibility, especially if there are no other staff available? This is a complex issue and there was no consensus on the issue in the thematic analysis.

### Issues with the current structure of IHEs

Many of the participants mentioned the relationship between the student IHE societies and the medical schools. Some remarked on the very definite separation of the two:

‘The (student IHE group name) charity and the tradition of going away to developing countries it totally separate from the School of Medicine. I think at one time it was said, “we acknowledge that you do this and we are very proud of you”, but as far as involvement, that’s where it ended with the school.’ P1

One participant wondered whether this ‘arms-length’ approach was taken by the medical schools in case there were mishaps while on IHE:

‘The faculty doesn’t have any real, umm not influence, but like they don’t have … governing say on anything (student IHE group name) does because they want to make sure it’s kept separate from the medical school in case there was any controversy or anything like that.’ P8

The status of students travelling on IHEs is often unclear. They fundraise using the name of the medical schools and the student IHE organizations. Many of the medical schools offer academic credits for time spent on IHE. Students in most of these schools must register their intentions to travel and have an assessment form completed and returned to the medical school after the IHE. The student societies are mentioned in many of the prospectuses of the medical schools, yet students are seen to travel quite independently of these institutions. One participant offered her opinion as to why things were this way:

‘The students were eager to keep it a student thing and the college themselves were eager to also keep it a student thing.’ P8

This leads to a situation where some of the student IHE groups try to provide support to their fellow students on IHE. One example was where an emergency e-mail address was set up in case of problems, but the students manning the account were themselves on IHE:

‘We said there was a (student IHE group name) e-mail, and we felt that, that was the best way because we didn’t know who would have Internet connection at the time. There wasn’t just one of us in charge of that, it was really any of the eight of us … I suppose maybe we were too lax in that because nothing’s happened yet with anyone on (student IHE group name) which is sad, you should be prepared I suppose.’ P7

One of the medical schools is currently working on putting IHEs on a more formal footing in the medical school. There are advantages and disadvantages to this from a student perspective:

‘I think it is important to make them a little official at least ……because sometimes you can be going to somewhere and you don’t know where you’re going really.’ P8

## Discussion

The personal and academic benefits of IHEs reported by participants in this research study have been previously reported in the literature [18–20]. While some participants felt supervision was lacking at times, others encountered excellent medical teachers who devoted much time and energy to facilitating the visiting students.

Difficulties with the distribution of charitable donations while on IHE have not been previously discussed in the literature. The creation of a benefactor-beneficiary relationship could negatively affect the successful learning experience. Current systems for the distribution of this money could be seen to reinforce historical inequities in developed-developing world relationships. Where students reported a lack of trust in host staff to manage any donations they brought on IHE, it is hard to see how they could have trusted the same staff to teach them medical knowledge and skills.

Is the donation of funds a way of offsetting the time and effort spent facilitating and hosting the IHE students? Some of the participants in the research study seemed to feel that was the case. They explained that they felt happier when there was a ‘transaction’ taking place, one participant called it a ‘symbiotic relationship’ (P7). This does, however, risk creating dependence by the hosts on these donated funds. If medical students decide not to travel to a particular clinic or health facility on IHE, no money is donated, and this may pose serious problems for the running of the host hospital.

Medical students do not have training or expertise in planning or managing projects in their own or other country’s health systems. They are often unaware of the many underlying issues that affect health systems in developing countries. Good health development practice would dictate the need for a full needs assessment before any visit and then shared planning of any improvements, with evaluation and monitoring taking place at every stage of the process [21]. It would make sense, therefore, that students should focus on gaining knowledge and skills on IHE rather than spending their time assessing how donations should be spent.

Ultimately, the issue of the sustainability of this type of erratic funding and the transparency of donations are issues for students, their IHE societies and their medical schools to address. If fundraising is to continue, perhaps the development of strong reciprocal arrangements with developing countrys’ medical schools would be a means of ensuring accountability and provide better educational encounters for students.

The impact of IHE experiences on medical students has not been widely discussed. There are a number of papers describing the ethical dilemmas faced by students, but they do not tend to look at the effects these have on the students after their return home [22, 23]. In this study it is clear that some of the events experienced by students on IHE had a profound effect on them. P1 talked about ruminating on the case of a child he was unable to treat effectively: ‘I hope the kid is still alive, that I didn’t do harm, even though I wasn’t able to help them’. These examples highlight issues of ethics, the need for pre-departure training, and formal debriefing after the IHE is completed.

The potential danger following troubling experiences like these is that if they are not discussed and reflected on in an effort to understand their root causes and outcomes, the student may be left with feelings of anger and dismay in relation to any or all attempts at improving health care in developing countries. Instead of the student returning from the IHE experience with a positive outlook on the possibility of improving global health and how this may be achieved, they may end up feeling disempowered with a very negative outlook.

The research study participants had a variety of opinions on what boundaries should be set on their activities while on IHE. Most, if not all, made it clear they would not do anything without supervision or being taught and encouraged to carry out new procedures or roles. After that, there was a divergence of opinions, with some students happy to take on new responsibilities, while others strictly limited themselves to what they would be allowed to carry out in their home country. P1 highlighted this turmoil very well when he explained: ‘It’s a setting of such limited resources I guess, it changes how much a medical student should do and it’s either that or absolutely nothing at all’. There is no easy answer to this dilemma. If students had time and space to discuss such issues and how to deal with them prior to going on IHE, this would give them some direction and the confidence to decline opportunities to act outside of their scope of practice. Ideally, clear advice from medical school staff would also be available during and after the IHE has taken place. There also needs to be a discussion among students and their medical schools on the difference between a learning experience and a volunteering experience. A medical student should go on IHE primarily to learn, whereas a volunteer has a certain level of qualification or skill and can function fairly independently in the clinical setting.

While student IHE societies are very keen to highlight their proud tradition of independence from the medical schools, some participants in this study had begun to realize that closer linkages with the medical schools for the conduct of safe and well-structured IHEs is likely to be in everyone’s best interest. While the student IHE groups carry out extensive preparation of the fundraising activities prior to departure, their disorganization and lack of any real contingency planning is notable. Despite the autonomous instincts of the students and their IHE groups, it would seem very sensible for medical schools to provide some basic level of pre-departure training, emergency planning and debriefing. Formalizing involvement of the medical schools may also allow for the running of IHEs to be more consistent and sustainable from year to year, as currently the committees of the student societies have little, if any, involvement with planning for the next year once they return to Ireland.

## Conclusion

There is much to be gained from IHEs, which can be truly worthwhile and formative experiences, but there is also the possibility of doing more harm than good. The proud history and enthusiasm for IHEs can lead to what one author referred to as a situation of ‘unexamined good intentions’ where it is presumed that this type of venture, with the aim of helping less fortunate people in distant parts of the world, is beyond scrutiny [24]. The issue of charitable donations as part of IHEs has not been addressed previously in the relevant literature. The fact that inadequate preparation prior to IHE can lead students to placing themselves in distressing situations is obvious and should be addressed. Irish medical schools need to recognize the need for formal pre-departure training and debriefing on return, and their role in the governance of funds raised by their students. The possibility of formal agreements with institutions in several countries, with a view to developing long-term sustainable partnerships should be explored where regular short-term rotations for students and staff, and possible reciprocal arrangements could be arranged. The whole structure and planning of IHEs should be reviewed by medical schools with the aim of standardizing them to maximize the quality of learning experiences. Consideration should be given to developing formal education and training for the medical students on norms of professionalism, standards of practice, cultural competence, dealing with conflict, and personal safety.

## Abbreviations

GMC: General Medical Council
HPM/CGH REC: Research Ethics Committee of Centre for Health Policy and Management & Centre for Global Health
IHE: international health elective
WEIGHT: Working Group on Ethics Guidelines for Global Health Training.
